# IL-6 and IL-10 are associated with good prognosis in early stage invasive breast cancer patients

**DOI:** 10.1007/s00262-017-2106-8

**Published:** 2017-12-18

**Authors:** Narmeen Ahmad, Aula Ammar, Sarah J. Storr, Andrew R. Green, Emad Rakha, Ian O. Ellis, Stewart G. Martin

**Affiliations:** 1 Division of Cancer and Stem Cells, Academic Clinical Oncology, School of Medicine, University of Nottingham, Nottingham University Hospitals NHS Trust, City Hospital Campus, Hucknall Road, Nottingham, NG5 1PB UK; 2 Division of Cancer and Stem Cells, Histopathology, School of Medicine, University of Nottingham, Nottingham University Hospitals NHS Trust, City Hospital Campus, Hucknall Road, Nottingham, NG5 1PB UK

**Keywords:** Breast cancer, IL-6, IL-10, Macrophage-associated cytokines, Metastasis

## Abstract

Macrophage-associated cytokines play an important role in cancer metastasis; however, the functions of interleukins (IL) 6 and 10 in breast cancer (BC) progression and metastasis are not clear. In this study the roles of IL-6/IL-10 in regulating vascular invasion and their prognostic significance in BC are investigated. MDA-MB-231 and MCF-7 migration (± IL-6 or IL-10) was assessed by scratch wound assay. Cancer cell adhesion to IL-6/IL-10 stimulated blood and lymphatic endothelial cells (EC) was investigated. Expression of IL-6 /IL-10 was assessed using immunohistochemistry in an annotated cohort of early stage BC (*n* = 1380) and associations with clinicopathological variables and clinical outcome evaluated. IL-6 did not alter BC cell migration however a dose-dependent inhibition in MDA-MB-231 migration with IL-10 treatment was observed (*P* = 0.03). BC cells were more adhesive to blood vs lymphatic EC, however, IL-6/IL-10 had no effect on adhesion patterns. High expression of IL-6/IL-10 was associated with clinicopathological criteria (e.g. hormone receptor status, all *P* < 0.05), improved disease-free survival (DFS; *P* < 0.05) and improved BC-specific survival (BCSS; only IL-6, *P* = 0.017). However, neither IL-6 nor IL-10 expression were independent prognostic factors from multivariate analysis. In BC subgroups, IL-6 and IL-10 were good prognosticators in terms of DFS in non-basal, non-triple-negative (non-TN), ER-positive, PgR-positive (only IL-10), and Her-2-negative (only IL-6) BC (all *P* < 0.05). IL-6 was associated with improved BCSS in non-basal, ER-positive and non-TN BC (all *P* < 0.05).

**Electronic supplementary material:**

The online version of this article (10.1007/s00262-017-2106-8) contains supplementary material, which is available to authorized users.

## Background

The presence of lymph node (LN) metastasis in breast cancer (BC) is associated with poor overall survival with recent studies showing that lymphatic vessel invasion (LI) rather than blood vessel invasion (BI) is the predominant form of lymphovascular invasion (LVI) in early stage invasive BC [1, 2]. Tumours with high densities of inflammatory infiltrate have a higher percentage of proliferating lymphatic vessels and LN metastasis [1, 3]. Macrophages represent a significant population of the inflammatory infiltrate and are linked to BC malignancy [4]. They can polarise into M1s, a pro-inflammatory anti-tumour type, or M2s, an anti-inflammatory form. The presence of inflammatory cytokines in the tumour milieu influences a number of processes at different stages of tumour progression, including initiation, proliferation, promotion, tumour cell conversion, angiogenesis, invasion, inhibition of apoptosis, immune surveillance, drug resistance and metastasis [5]. Comparatively little is known, however, about the roles of the macrophage-associated cytokines Interleukin-6 (IL-6) and interleukin-10 (IL-10) in the regulation of LVI, and LN metastasis or even their expression in breast tumours.

IL-6 is a pleiotropic cytokine that plays important roles in immune response, inflammation, and haematopoiesis. It is produced by a variety of normal cells including monocytes and macrophages [6], but is also expressed by multiple tumour tissue types, such as breast, prostate, colorectal and ovarian cancer [7–10]. IL-6 may also play an important role in various aspects of tumour behaviour, including apoptosis, tumour growth cell proliferation, migration and invasion, angiogenesis and metastasis [11].

IL-10, initially termed ‘cytokine synthesis inhibitor’ or ‘cytokine inhibitory factor’ due to its inhibitory action on cytokine production by T helper cells, is produced by almost all leukocytes, as well as numerous human tumour cells including breast, kidney, colon, pancreas, malignant melanomas and neuroblastomas [12–17]. It belongs to the IL-10 family of cytokines and plays a role in the pathogenesis of infectious disease and inflammation. IL-10 is essential to suppress tumour promoting inflammation mediators (reviewed in [18]); however, IL-10 might play a potential role in regulating tumour angiogenesis [19].

Both IL-6 and IL-10 signal through the signal transducer and activator of transcription 3 (STAT3) [20]. Although signalling mainly through STAT3, IL-6 is pro-inflammatory whereas IL-10 is anti-inflammatory and suppresses the expression of other cytokines by immune cells. Such responses have been explained in dendritic cells by temporal activation of STAT3 by IL-6 vs prolonged effect of IL-10 via suppressor of cytokine signalling-3 (SOCS3) activation [20].

The role of the macrophage-associated cytokines IL-6 and IL-10 in LVI or LN metastasis has not been previously addressed. There is little information available about the in vitro effect of IL-6 and IL-10 on the phenotypic behaviour of BC cells in terms of tumour cell migration or adhesion to lymphatic and blood endothelium, which are key steps in the metastatic process. Equally, the prognostic significance of BC tissue expression of these cytokines has not been previously investigated in a large cohort of patient samples. The aim of this study was to assess the effect of IL-6 and IL-10 on BC cell migration and endothelial adhesion, examining for differential effects on blood vs lymphatic endothelium, as well as assessing the prognostic significance of IL-6 and IL-10 expression in a large cohort of BC patients.

## Methods

### Cell lines and culture

BC cell lines MCF-7 (luminal phenotype) and MDA-MB-231 [basal/triple negative (TN) phenotype], human microvascular endothelial cells hMEC-1 (passage window 4–18), human telomerase reverse transcriptase immortalised lymphatic EC (hTERT-LEC, passage window 27–34) [21] were used in this study. BC cell lines were used across a 10-passage window. MCF-7 cells were cultured in RPMI supplemented with penicillin/streptomycin (100 U/mL and 0.01 mg/mL, respectively) and 10% iron supplemented donor bovine serum (DBS, Gibco). MDA-MB-231s were cultured in MEM supplemented with l-glutamine (2 mM), non-essential amino acids (0.1 mM) and 10% iron-supplemented DBS. hMEC-1 were grown in EMB-2 (Lonza) containing EGF (0.01 µg/mL), hydrocortisone (5 µg/mL, Sigma), penicillin/streptomycin and 10% iron-supplemented DBS; and hTERT-LEC grown in EGM-2MV kit (Lonza). All cells were mycoplasma free and tumour cell line authentication conducted by short tandem repeat verification (PowerPlex 16, Promega).

### Scratch wound migration assay

Methodology was described in detail elsewhere [22] with the following concentrations of cytokines being used to stimulate tumour cells (IL-6 at 2.5, 5, or 10 ng/mL; or IL-10 at 5, 10, or 15 ng/mL, Peprotech). Wound closure was measured using photomicrographs taken at 0, 2, 4, 6, and 24 h at × 100 magnification. The percentage reduction of the scratch area at different time points represented the level of cellular migration and was measured using ImageJ 1.46e (National Institute of Health, USA). Experiments were conducted three times, each in triplicate.

### Static adhesion assay

Assay methodology was described in detail previously [22]. Briefly, a confluent endothelial monolayer remained unstimulated, or was stimulated for 24 h either with IL-6 (2.5, 5 or 10 ng/mL) or IL-10 (5, 10, or 15 ng/mL). Tumour cells were fluorescently labelled with 1 µM of Cell Tracker Green CMFDA (Invitrogen) for 30 min at 37C, then 1 × 10^5^cells/well (24-well plate) added for 35 min. Non-adherent tumour cells were washed and adherent cells counted using a fluorescence microscope (Nikon) in two fields of view/well at × 100 magnification. Experiments were conducted three times, each in duplicate. As a positive control, adhesion of peripheral blood mononuclear cells (PBMCs, isolated from whole blood using a density gradient centrifugation method) to EC stimulated with and without 5 ng/mL TNF-α (Peprotech) for 24 h was always performed directly prior to tumour cell adhesion assay to assess the responsiveness of EC to cytokine stimulation as described previously [22].

### Patient samples

A total of 1380 patients with early stage invasive BC, treated at Nottingham University Hospitals between 1988 and 1998 with long-term follow-up, were included. Data on a wide range of clinicopathological markers, receptor status, and many different biomarkers including LVI have been described previously [1, 2]. The median age of patients was 55 years (ranging from 18 to 70 years). Patients were managed under a uniform protocol, where all underwent mastectomy or wide local excision followed by radiotherapy. The clinicopathological features of the patients and tumours used in the analysis are shown in Table 1.

**Table 1:** Clinicopathological characteristics of patients (*n* = 1380)

Clinical features	Number (%)
Age (years)	
≤ 40	119 (8.6)
> 40	1261 (91.4)
Stage	
I	829 (60.1)
II	418 (30.3)
III	125 (9.1)
ND	8 (0.6)
LI	
Negative	752 (54.5)
Positive	383 (327.8)
ND	245 (17.8)
NPI	
Good (< 3.4)	418 (30.3)
Moderate (3.4–5.4)	696 (50.4)
Poor (> 5.4)	255 (18.5)
ND	11 (0.8)
PgR status	
Negative	532 (38.6)
Positive	774 (56.1)
ND	74 (5.4)
Basal like status	
Non-basal	1026 (74.3)
Basal like	268 (19.4)
ND	86 (6.2)
Recurrence	
No	945 (68.5)
Yes	426 (30.9)
ND	9 (0.7)
Size (cm)	
≤ 2	824 (61.0)
< 2	529 (38.3)
ND	9 (0.7)
Grade	
I	235 (17)
II	468 (33.9)
III	668 (48.3)
ND	9 (0.7)
BI	
Negative	705 (51.5)
Positive	3 (0.2)
ND	672 (48.7)
ER status	
Negative	337 (24.4)
Positive	1002 (72.6)
ND	41 (3.0)
Her-2 status	
Negative	1172 (84.9)
Positive	184 (13.3)
ND	24 (1.7)
TN status	
Non-TN	1114 (80.7)
TN	233 (16.2)
ND	43 (3.1)
Distant metastasis	
No	945 (68.5)
Yes	426 (30.9)
ND	9 (0.7)

*LI* lymphatic vessel invasion, *BI* blood vessel invasion, *NPI* Nottingham Prognostic Index, *ER* oestrogen receptor, *PgR* progesterone receptor, *Her-2* epidermal growth factor receptor 2, *TN* triple negative, *ND* not determined

BC-specific survival (BCSS) was defined as the time interval (in months) between the start of primary surgery to death resultant from BC. Disease-free survival (DFS) time was defined as the time interval (in months) between the primary surgery and first recurrence of cancer. The mean survival time of the cohort of patients was 225.4 months. This study is reported in accordance with REMARK criteria [23]. Ethical approval was granted by Nottingham Research Ethics Committee 2 under the title ‘Development of a molecular genetic classification of breast cancer’ (C202313) and by Nottingham Research Ethics Committee under the title ‘Blood tumour markers in breast cancer’.

### Immunohistochemistry (IHC)

Information on tissue microarray (TMA) construction is provided elsewhere [24]. Freshly cut 4-μm formalin-fixed paraffin-embedded breast cancer TMA sections were deparaffinised and rehydrated using xylene, industrial methylated spirit and water. Antigen retrieval was performed by microwaving samples in citrate buffer (pH = 6) at 750 W for 10 min, followed by 10 min at 450 W. Endogenous peroxidase activity was blocked with 10% H_2_O_2_ in methanol for 10 min, followed by treatment with normal horse serum (1:50, Vector laboratories) for 30 min. Polyclonal goat anti-IL-6 (1:25, R&D systems) or goat anti-IL-10 (1:100, R&D systems) were then incubated for 1 h at room temperature with the tissues. Secondary antibody was added for 1 h, followed by treatment with Vectastain Goat Elite ABC kit (Vector Laboratories) for 30 min. DAB substrate was added and slides were counter-stained with haematoxylin. Tonsil sections were used as a positive control. For negative controls, the staining protocol was performed, but primary antibody omitted.

IL-6 and IL-10 antibody specificity was confirmed through peptide blocking experiments. Anti-IL-6 or anti-Il-10 antibody was neutralised with recombinant human IL-6 (rIL-6, 1 µg) or rIL-10 (2 µg) (Peprotech) overnight at 4 °C and the same staining protocol then carried out as above. TMA stained slides were scanned using a Nanozoomer Digital Pathology scanner (Hamamatsu Photonics) at × 200 magnification. H-scores were calculated by multiplying the percentage area scoring positive by the respective intensity using the following formula: (% of cells stained weak × 1) + (% of cells stained moderate × 2) + (% of cells stained strong × 3) (range 0–300) [25]. The core was considered assessable if tumour cells were present in > 40% of its total area. 30% of cores were examined by a second independent assessor blinded to scores and clinicopathological data with good concordance between both scorers (single measurement intra-class correlation of 0.816 for IL-6 and 0.714 for IL-10). Immunohistochemical scores were dichotomised based on BCSS analysis using X-tile software (a free bioinformatics-based tool developed by Yale University to provide cut-points in an independent and an unbiased way) [26].

### Statistical analysis

Data are presented as mean ± standard deviation (SD) for adhesion and migration endpoints. A one-way ANOVA test was used to assess significance between the different cytokine concentrations at a given time point followed by a paired *t* test when the ANOVA test showed significance. For immunohistochemistry, the relationship between categorised protein expression and clinicopathological data was measured using the Pearson Chi-squared test of association (*χ* ^2^) or Fisher’s exact test. Pearson correlation was used to assess the correlation between IL-6 and IL-10 expression, and the Spearman’s rank correlation test was used to assess correlation between IL-6/IL-10 and STAT3 expression (assessed previously [27]). Survival curves were plotted using the Kaplan–Meier (KM) method and the statistical significance between groups determined by the log-rank test. Multivariate survival analysis was performed by Cox proportional hazards analysis with data adjusted to include potential confounding factors that were individually significantly associated with survival from KM analysis and log-rank test. *P* < 0.05 was considered statistically significant. Statistical analysis was performed using SPSS 22.0 software (IBM SPSS Statistics).

## Results

### IL-6 has no significant effect on cell migration

**Fig. 1:**
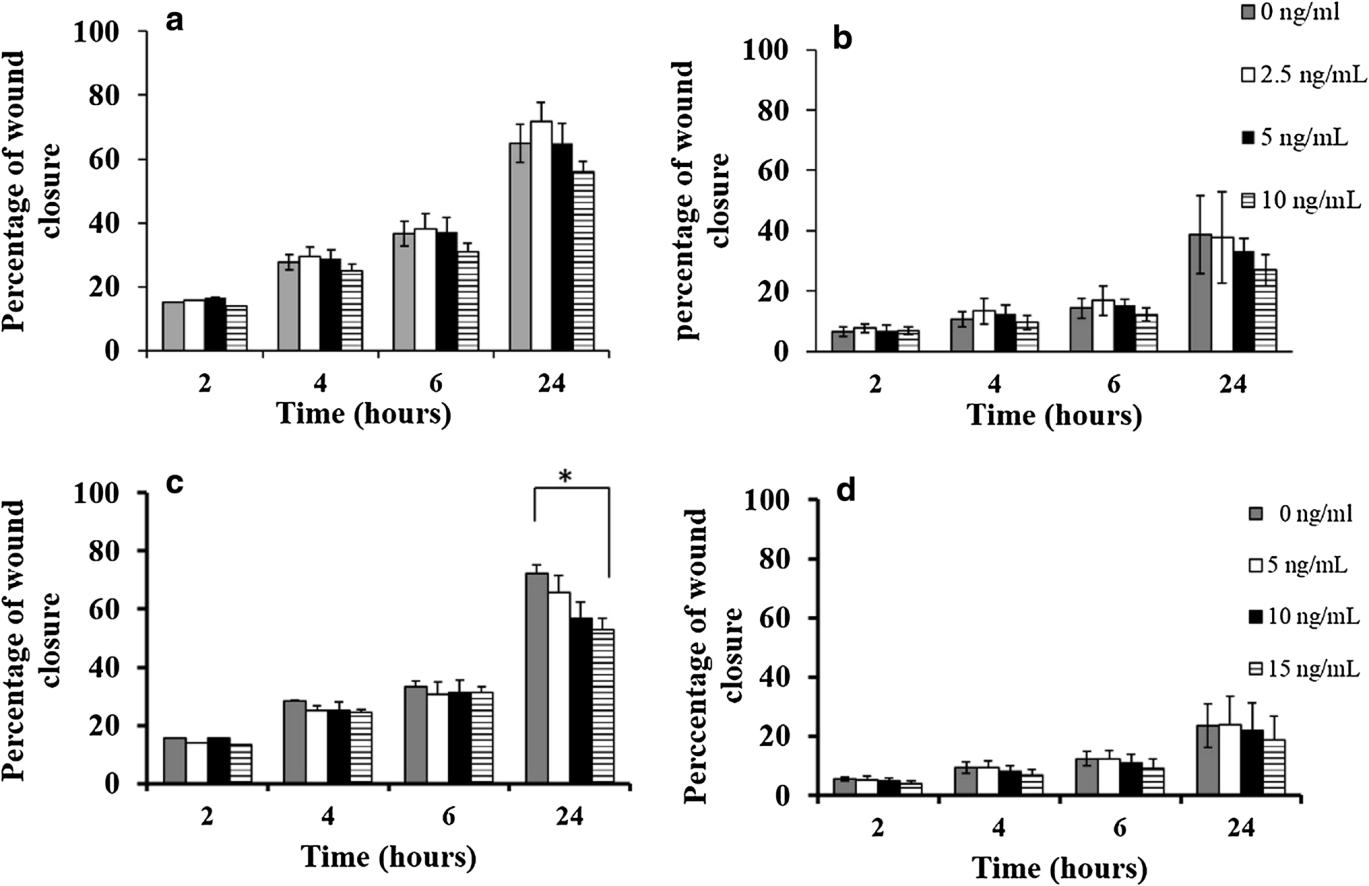
Effect of recombinant human IL-6 or IL-10 stimulation on breast cancer cell migration rate. IL-6 treatment caused a slight decrease in MDA-MB-231 (**a**) and MCF-7 (**b**) migration after 24 h treatment; however, the reduction in migration did not reach statistical significance. IL-10 (15 ng/mL) was associated with a significant reduction in MDA-MB-231 migration (*P* = 0.03) (**c**), but has no significant effect on MCF-7 migration (**d**). Data are presented as mean ± SD of three independent experiments each carried out in triplicate, and *P* values evaluated by paired sample *t* test (asterisk represent a significant *P* value)

Under unstimulated conditions, the migration of MDA-MB-231 was higher than that of MCF-7 cells (percentage wound closures were 65 ± 9 vs. 39 ± 13%, respectively, at 24 h). After 24 h treatment, low doses of IL-6 (2.5 ng/mL) caused a slight increase in MDA-MB-231 migration in comparison to unstimulated cells, whereas higher doses (i.e. 5 and 10 ng/mL) were associated with slower migration but no statistical significance was observed (*P* = 0.27, ANOVA test) (Fig. 1a). Similarly, IL-6 treatment caused a marginal, but non-significant, decrease in MCF-7 migration at 24 h treatment (*P* = 0.51, ANOVA test, Fig. 1b).

### IL-10 has an inhibitory effect on migration in vitro

A dose-dependent decrease in MDA-MB-231 migration was seen with increasing concentrations of IL-10, with a weak but significant difference seen at 15 ng/mL in comparison to control (*P* = 0.03). The percentage wound closure at 24 h was 63 ± 7, 60 ± 6, and 56 ± 4% when cells were treated with 5, 10, and 15 ng/mL, respectively, compared to 70 ± 3% wound closure with control cells (Fig. 1c). The percentage closure with MCF-7 cells, at 24 h post wounding, was 24 ± 10, 22 ± 10, and 19 ± 8% when cells were treated with 5, 10, and 15 ng/mL compared to control, at 23 ± 7% (Fig. 1d).

### IL-6 and IL-10 do not affect tumour–endothelial cell adhesion

**Fig. 2:**
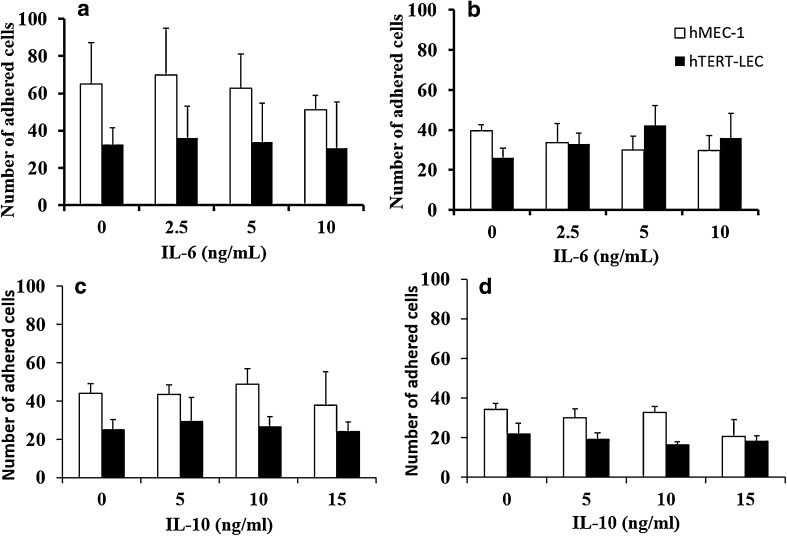
Effect of IL-6 and IL-10 on endothelial adhesion patterns of MDA-MB-231 and MCF-7. IL-6 or IL-10 stimulation of blood (hMEC-1) and lymphatic (hTERT-LEC) endothelial cells did not significantly alter adhesion patterns compared to the unstimulated controls. **a** MDA-MB-231 and **b** MCF-7 adhesion to IL-6 stimulated endothelial cells, **c** MDA-MB-231 and **d** MCF-7 adhesion to IL-10 stimulated endothelia. Data represent the mean of adhered cells ± SD of three independent experiments, each carried out in duplicate (*n* = 6)

As observed previously [22] tumour cells show a preference for adhesion to blood rather than lymphatic endothelium. Both MDA-MB-231 and MCF-7 adhesion to hMEC-1-was approximately 50% higher than adhesion to hTERT-LEC under unstimulated conditions (Fig. 2) with MDA-MB-231 showing higher affinity than MCF-7 to EC. Pre-stimulation of hMEC-1 or hTERT-LEC with either IL-6 or IL-10 did not significantly alter tumour cell adhesion patterns when compared to unstimulated conditions (Fig. 2).

### Expression of IL-6 and IL-10 in breast tumour specimens

**Fig. 3:**
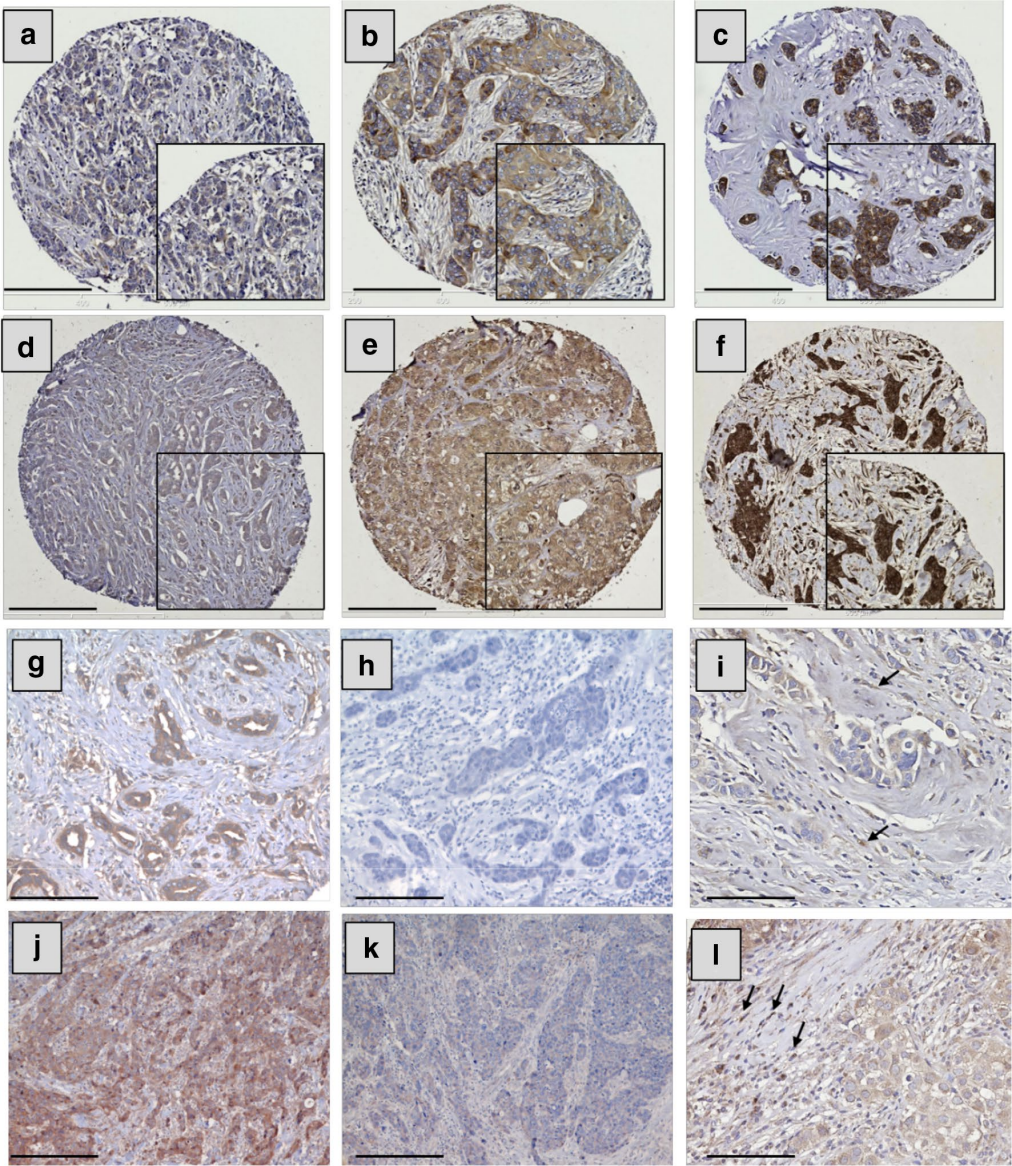
IL-6 and IL-10 expression in breast cancer and stromal cells. Representative images of tumour staining with IL-6 (**a**–**c**) and IL-10 (**d**–**f**). Staining pattern: **a, d** weak, **b, e** moderate, and **c, f** strong. **g, h, j, k** Representative images of specificity tests of IL-6 and IL-10 antibodies, respectively: BC staining with IL-6 antibody (**g**) or IL-6 antibody blocked overnight with 1 µg of rIL-6 (**h**); BC staining with IL-10 antibody alone (**j**) or IL-10 antibody blocked overnight with 2 µg of rIL-10 (**k**). Examples of stromal expression of IL-6 and IL-10 are shown in **i, l**, respectively (black arrows). Photomicrographs: **a**–**f**; × 100 and **g**–**l** × 200 magnification; inset boxes at × 200 magnification; scale bars 100 µm

Both IL-6 and IL-10 showed positive cytoplasmic staining in BC cells with a heterogeneous staining pattern between, as well as within, certain tumour cores varying from weak to intense. Representative photomicrographs of staining intensity of IL-6 and IL-10 expression in BC are shown in Fig. 3a–f. The specificity of IL-6 and IL-10 antibodies was confirmed using blocking rIL-6 or rIL-10 as shown in Fig. 3g, h, j, k, respectively. In certain specimens, expression of these cytokines was observed in a subset of inflammatory cells but this expression was not quantified (Fig. 3i, l, respectively).

The median H-scores for IL-6 (*n* = 1191) and IL-10 (*n* = 878) expression were 80 (ranging between 0 and 275) and 170 (ranging between 60 and 265), respectively. IL-6 cut-point for stratification was 95, with 349 (29.3%) cases showing high expression. The IL-10 cut-point was 180 with 150 (17.1%) with high expression. The expression of IL-6 and IL-10 were positively and significantly correlated (Pearson correlation, *r* = 0.279, *P* < 0.001, *n* = 689). IL-6 was positively correlated with nuclear (*r* = 0.142, *P* < 0.001, *n* = 836) and cytoplasmic (*r* = 0.292, *P* < 0.001, *n* = 852) STAT3. IL-10 was similarly positively correlated with nuclear and cytoplasmic STAT3 (*r* = 0.221, *P* < 0.001, *n* = 643 and *r* = 0.235, *P* < 0.001, *n* = 655, respectively).

Dichotomised data were tested for associations with clinicopathological criteria (Table 1). There was no significant relationship between IL-6 expression and either stage, LN involvement or LVI that had been previously assessed by immunohistochemistry [1–3]. However, high IL-6 expression was significantly associated with patients over 40 years (*P* = 0.033), lower tumour size (*P* = 0.006), lower tumour grade (*P* = 0.001), lower Nottingham Prognostic Index (NPI) scores (*P* < 0.001), positive oestrogen receptor (ER) status (*P* = 0.046), and positive progesterone receptor (PgR) status (*P* < 0.001) (Table 2). As with IL-6 there was no significant relationship between IL-10 with either stage, LN metastasis or IHC determined LVI (Table 2). High IL-10 expression was, however, significantly associated with lower tumour grade (*P* < 0.001), low NPI value (*P* < 0.001), positive ER (*P* < 0.001), positive PgR (*P* < 0.001), negative Her-2 (*P* = 0.003) as well as non-TN status (*P* = 0.003).

**Table 2:** Association between IL-6/IL-10 expression and clinicopathological variables

Variable	IL-6 expression (*n* = 1191^a^)			IL-10 expression (*n* = 878^a^)		
	Low	High value	*P* value (*X* ^2^ value)	Low	High	*P* value (*X* ^2^ value)
Age (years)						
≤ 40	83	21	**0.033** (4.566)	73	10	0.22 (1.164)
> 40	759	328		655	140	
Size (cm)						
≤ 2	490	232	**0.006 (7.576)**	424	94	0.361 (0.833)
> 2	348	114		299	56	
Tumour stage						
I	488	223	0.139 (3.946)	405	96	0.177 (3.466)
II	270	95		245	40	
III	80	28		74	14	
Tumour grade						
I	118	77	**0.001 (13.326)**	114	38	< **0.001 (29.247)**
II	277	115		227	69	
III	443	154		382	43	
NPI						
< 3.4	218	127	< **0.001 (16.140)**	192	67	< **0.001 (19.741)**
3.4–5.4	445	169		372	61	
> 5.4	174	49		158	22	
Basal status						
Non-basal	628	259	0.783 (0.076)	542	119	0.205 (1.605)
Basal	160	69		143	23	
ER status						
Negative	221	72	**0.046 (3.984)**	201	21	< **0.001 (12.895)**
Positive	598	265		502	126	
PgR status						
Negative	360	103	< **0.001 (19.688)**	310	36	< **0.001 (17.349)**
Positive	437	230		383	104	
Her-2 status						
Negative	710	298	0.762 (0.092)	606	138	**0.003 (8.588)**
Positive	116	46		109	9	
Triple negative (TN)						
Non-TN	671	291	0.062 (3.483)	570	131	**0.003 (8.868)**
TN	149	46		136	13	
LI						
Negative	457	197	0.287 (1.123)	385	87	0.192 (1.701)
Positive	250	92		211	36	
Positive	3	0		2	1	
LN status						
Negative	431	197	0.136 (2.225)	362	85	0.109 (2.566)
Positive	307	114		296	51	

Data are presented as absolute numbers. *P* values are resultant from Pearson *χ* ^2^ test of association, with significant values indicated in bold

*NPI* Nottingham Prognostic Index, *ER* oestrogen receptor, *PgR* progesterone receptor, *Her-2* epidermal growth factor receptor 2, *LI* lymphatic vessel invasion

^a^Numbers of analysed cases are different from total number of patients due to random core dropout during IHC staining process. No associations were performed with BI due to limited number of BI positive cases (*n* = 3, Table 1)

The median values of IL-6 and IL-10 expression (80 and 170, respectively) were also used as cut-point values to dichotomise the data into high and low expression of the cytokine. Using the median as a cut-point and Mann–Whitney or Kruskal–Wallis as a statistical test, similar results were obtained to those observed with the X-tile cut-point values. IL-6 was significantly associated with younger age and smaller tumour size (*P* = 0.002 and *P* = 0.007, respectively) and both IL-6 and IL-10 were significantly associated with lower grade (*P* < 0.001 and *P* = 0.001, respectively), lower NPI (both P < 0.001), positive ER and PgR status (all *P* < 0.001, respectively) and with non-TN status (both *P* < 0.001). High IL-10 expression was also associated with negative Her-2 status (*P* = 0.003).

### High IL-6/IL-10 expression is associated with improved survival

**Fig. 4:**
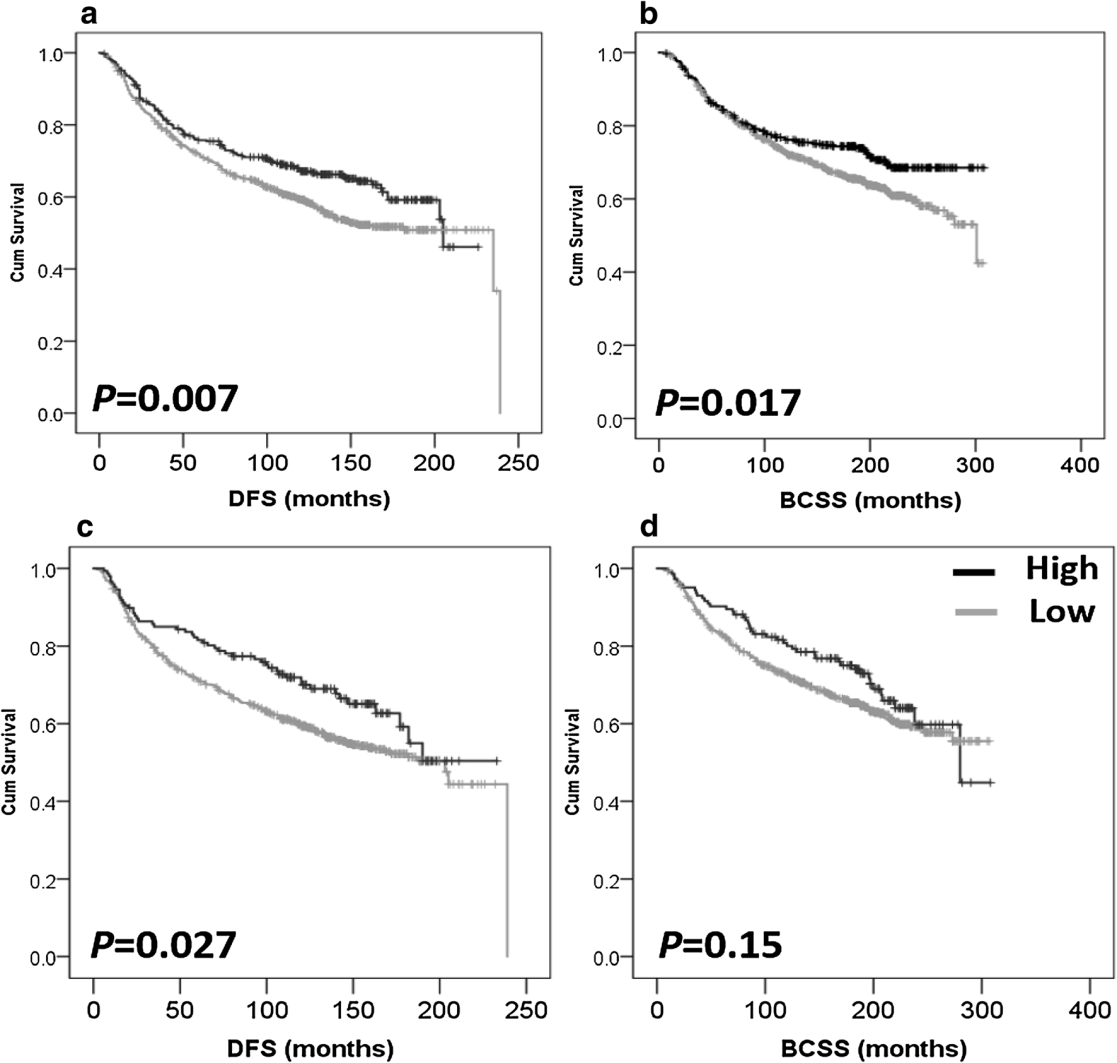
Kaplan–Meier analysis of association between IL-6 and IL-10 expression with breast cancer prognosis. High IL-6 expression is significantly associated with improved disease-free survival (*P* = 0.007, **a**) and improved breast cancer-specific survival (*P* = 0.017, **b**). High IL-10 expression is significantly associated with improved disease-free survival (*P* = 0.027, **c**) but not breast cancer-specific survival (*P* = 0.150, **d**). Significance was determined using the log-rank test. Black represents high expression and grey represents low expression of the cytokine

Survival analyses show that high IL-6 expression is associated with better DFS (*P* = 0.007) (Fig. 4a). The mean DFS was 158.2 months in patients with high IL-6 in comparison to 151.268 months in patients with low expression of IL-6. IL-6 expression was also associated with better BCSS (*P* = 0.017) (Fig. 4b) with a mean of 238.071 versus 218.742 months survival time in the high and low IL-6 groups, respectively. Similarly, high IL-10 expression was associated with better DFS (*P* = 0.027) (Fig. 4c) and the mean DFS time was 166.132 months for high IL-10 in comparison to 150.336 months in patients with low IL-10 expression. However, IL-10 expression was not associated with BCSS (*P* = 0.150) (Fig. 4d). In multivariate Cox regression analysis the following factors were included in the analysis: patient age, tumour size, tumour stage, tumour grade, NPI, ER, PgR and Her-2 status, LVI, and LN status (all were significant with regard to survival analysis with *P* values < 0.001 for all markers in terms of BCSS (except for age, *P* = 0.03), and *P* < 0.001 for all markers in terms of DFS [except for age and ER status where *P* values were 0.002 and 0.004, respectively)]. Expression of IL-6 was not an independent prognostic factor for DFS [hazard ratio (HR) = 0.788; 95% confidence interval (CI) = 0.603–1.029; *P* = 0.08], or BCSS (HR = 0.852; 95% CI = 0.627–1.157; *P* = 0.305) (Supplementary Table 1). Similarly, IL-10 expression was not an independent prognostic factor for DFS (HR = 0.799; 95% CI = 0.543–1.175; *P* = 0.254) (Supplementary Table 1).

Survival analysis of IL-6 and IL-10 expression based on median values as cut-points did not show prognostic significance for either cytokine in terms of BCSS and DFS (all *P* values were > 0.05, data not shown).

### IL-6/IL-10 expression and survival in breast cancer subgroups

Survival analysis was performed in basal-like (negative for ER, PgR and Her-2 and positive for cytokeratins CK5/6 and CK14 and/or EGFR [28]) and non-basal phenotype, and in receptor-positive and receptor-negative disease subgroups to assess the significance of IL-6 and IL-10 expression in terms of DFS and BCSS.

High IL-6 expression was significantly associated with DFS in non-basal (*P* = 0.004), non-TN disease (*P* = 0.003), ER-positive (*P* = 0.025), and Her-2-negative (*P* = 0.026) BC (Supplementary Fig. 1a–d, respectively). IL-6 expression was not associated with survival of BC patients with basal-like disease (*P* = 0.189), ER-negative (*P* = 0.212), PgR-positive (*P* = 0.103), PgR-negative (*P* = 0.056), Her-2-positive (*P* = 0.074) nor with the TN (*P* = 0.687) BC. IL-10 expression was also associated with better DFS in non-basal samples (*P* = 0.011), non-TN (*P* = 0.015), ER-positive (*P* = 0.039), and PgR-positive (*P* = 0.029) (Supplementary Fig. 2a–d, respectively) cancers but not with the basal (*P* = 0.980), ER-negative (*P* = 0.801), PgR-negative (*P* = 0.835), Her-2-positive (*P* = 0.144), Her-2-negative (*P* = 0.135), or TN (*P* = 0.760) samples. The observed significance with different subgroups was not maintained in multivariate analysis (data not shown).

High IL-6 expression was significantly associated with better BCSS in non-basal-like phenotype (*P* = 0.008) non-TN (*P* = 0.002) and ER-positive disease (*P* = 0.041) (Supplementary Fig. 3a–c, respectively) but not with the other subgroups. No significant associations were seen between IL-10 expression and BCSS in any of the histopathological subgroups.

The co-expression of IL-6 and IL-10 in the total cohort of patients was grouped into four categories (high IL-6/high IL-10, high IL-6/low IL-10, low IL-6/high IL-10, low IL-6/low IL-10, *n* = 689). DFS and BCSS of patients in the four different groups was analysed, however, no significant associations were obtained (DFS: *P* = 0.206 and BCSS: *P* = 0.249, data not shown).

## Discussion

Previously published data regarding the role of IL-6 and IL-10 in BC mostly investigated levels in serum or in whole tissue extracts without focusing on tumour tissue localisation. Expression of IL-6 and IL-10 (macrophage-associated cytokines) in tumour tissue has only been determined in a few studies and using relatively small patient cohorts [7–9, 19, 29–32] with limited information about prognostic significance (e.g. 108 invasive BC cases stained for IL-6 and other cytokines [33]). The current study aimed to investigate the potential role(s) that IL-6 and IL-10 may play in the metastatic process in vitro and examine IL-6 and IL-10 expression in BC tissues to determine their association with clinicopathological parameters and prognostic significance.

IL-6 has been shown to increase T47D and MDA-MB-231, but not MCF-7, transmigration when used as a chemoattractant in Boyden Chamber-based assays (IL-6 concentrations used ranged between 10 and 200 ng/mL) [34]; however, the direct stimulation of BC cell lines with IL-6 did not alter the migratory ability of MDA-MB-231 and MCF-7 cells in the current study. The effect of IL-6 on cell migration may be cell-type dependent and varies between in vitro and in vivo models of BC [35]. The current in vitro data support the findings of the IHC data where no association was found between IL-6 expression and LN metastasis or LVI positivity.

Little is known about the effect of IL-10 on human cancer cell line migration. Previous studies showed that migration and invasion of HT-29 cell line was not significantly changed following treatment with IL-10 [36], however, in murine models of breast and melanoma cancers IL-10 showed anti-metastatic effects [37]. The effect of rIL-10 on BC cell migration and invasion has not been previously reported. Results of the current study suggest that IL-10 may inhibit migration of MDA-MB-231 in a dose-dependent manner. A similar trend was seen with MCF-7 migration, which was slightly decreased with IL-10 treatment for 24 h, albeit non-significant. The control migration rate of MCF-7 was slower than MDA-MB-231. Therefore, it is possible that MCF-7 cells may require a longer migration follow-up time (e.g. 48–72 h) to observe significant inhibition of migration following IL-10 treatment. The inhibitory effects of IL-10 on tumour progression have previously concentrated on the anti-tumour immune effects; one of the suggested mechanisms is via inducing infiltration and activation of cytotoxic CD8 cells [38]. In contrast to this proposed role, IL-10 production is associated with T cell inactivation and impairment of adaptive immunity [39] via a direct effect on Th17 and Th17 and Th1 cells [40]. The complex role IL-10 plays in determining the immune response seems to be dependent on the tissue microenvironment and the expression of IL-10 receptors on different types of immune cells [18].

Previous data suggested that IL-6 is associated with metastasis and the stromal IL-6 expression is key for this process [35, 41]. The effect of EC stimulation with IL-6, and IL-10, on tumour–endothelial cell adhesion was investigated in this study. Tumour cell adhesion patterns to both IL-6 and IL-10 stimulated hMEC-1 and hTERT-LEC were unaltered. Similar results have been reported with pancreatic carcinoma cells adhesion to hMEC-1 stimulated with IL-6 [42]; however, as far as we are aware no published data is available investigating the effect of IL-10 on tumour cell adhesion.

The significance of IL-6 and IL-10 expression in BC was further investigated in a large cohort of well characterised early stage invasive BC patients with long-term clinical follow-up. Previous studies looking at expression of IL-6 and IL-10 showed similar cytoplasmic expression patterns in tumour cells of the breast and other tumour types [7–9, 19, 29–33]. IL-10 expression has been shown to associate with improved survival rates of patients with colorectal cancer [32] and BC [43], but with poor survival in non-small cell lung cancer [44] and gastric cancer [19]. IL-6 has been linked with malignancy across a number of different tumour types [10, 29, 34], with its importance being related to downstream signalling via STAT3 activation. Similar to current results, nuclear expression of phosphorylated STAT3 (p-STAT3, the activated form of STAT3) has also been recently shown to be associated with small tumour size, low grade and negative LVI and to be a positive prognosticator of BCSS [27]. When the relationship between the expression of IL-6/IL-10 and p-STAT3 was examined there was a positive correlation between IL-6/IL-10 and STAT3 expression. Moreover, there was also a positive correlation between IL-6 and IL-10 expression. It should be remembered, however, that such statistical correlations are based on protein expression rather than functional assays looking at activation of STAT3 by IL-6/IL-10. The conflicting data regarding the role of IL-6 may suggest further consideration being given to investigating IL-6 mediated and IL-6 inhibitory pathways in BC.

In the current study, high IL-6 expression was associated with good prognostic variables, i.e. lower tumour size, lower grade, and lower NPI value. Some of these results were in accordance of others, e.g. low grade and ER-positive tumours had high IL-6 expression [30] but disagree with others, e.g. Chavey et al., using whole BC tissue lysates, showed an inverse association between BC expression of IL-6 and IL-10 and ER positivity [29]. The difference in IL-6 assessment between the current study and Chavey’s study (i.e., tumoural vs. whole tissue lysates) may in part explain the difference in the results. By using tissue lysate the expression of IL-6 by stroma will be representative of total rather than tumoural expression of IL-16. Interestingly, there is evidence suggesting that ER activation inhibits STAT3 signalling in BC cell lines [45]. In the current study, high IL-6 expression is associated with ER positivity. It is possible that ER active signalling may inhibit autocrine downstream signalling in tumour cells but that it is still maintained in stromal cells. In those cancer types where IL-6 has been shown to play a pro-tumoural effect, it may well be that the ER pathway is less important than it is in BC. In vivo information from murine models of TN-BC showed different response to STAT3 pathway blocking in vitro vs in vivo (i.e. IL-6 pathway inhibition did not influence tumour cell proliferation in vitro but potently reduced tumour growth using TN models of murine BC in vivo) [35]. Therefore, an investigation using in vivo models of ER-positive/negative tumours is warranted.

The lack of a significant relationship between IL-6 expression with LN metastasis is in accordance with a previous BC patient study (*n* = 149) [30]. However, a significant association between IL-6 (total expression in tumour and stroma) and negative LN status has recently been reported [33] and such results support IL-6’s association with good prognostic markers in BC.

In terms of IL-6 survival analysis, high expression was associated with improved DFS and BCSS; similar to what has been found with IL-6 mRNA levels in BC [10], however IL-6 was not an independent prognostic factor. A recent study has shown that high IL-6 expression was an independent prognostic marker in terms of longer overall survival and DFS in BC [33], however the difference in sample size (*n* = 108 vs. 1191 in the current study) and the prognostic factors included in Cox regression analysis may explain the differences between the Fernandez-Garcia et al. paper [33] and current results. Furthermore, current results of the prognostic significance of IL-6 in BC phenotypic subgroups suggest that longer BCSS and DFS are mainly related to patient groups with better prognosis (i.e. non-basal-like, non-TN, ER-positive).

In the current study, a strong association was found between high IL-10 and lower tumour grade, lower NPI level, positive ER and PgR status, negative Her-2 expression as well as with the non-TN type. DFS and BCSS analysis of IL-10 expression in BC phenotypic subgroups suggested that high IL-10 expression was a marker of better prognosis in ER-positive, non-basal-like, non-TN. In the total cohort, IL-10 was also significantly associated with DFS, which may support the in vitro data where high levels of rIL-10 caused a decrease in MDA-MB-231 migration. However, tumour expression of IL-10 was not a prognostic factor in terms of BCSS. Moreover, as for IL6, IL10 was not an independent prognostic factor.

Survival analysis of IL-6/IL-10 co-classification was not associated with prognosis. The co-expression of three cytokines (i.e. IL-6/IL-10 with IL-1) was recently analysed against survival endpoints in BC [33]; however, only stromal expression, rather than tumoural expression, of the combined groups showed prognostic significance. Such results suggest that IL-6 and IL-10 may not have a prognostic significance in BC, but their function in modulating the tumour microenvironment and altering cancer cell motility and, perhaps, metastatic ability, requires further investigation.

## Conclusion

Results presented here provide an insight into the role of IL-6 and IL-10 in BC progression. Results demonstrate that high concentrations IL-10 can reduce in vitro migration, but do not influence adhesion to blood or lymphatic cells. High expression of IL-6 or IL-10 in BC tissues is significantly associated with some clinicopathological criteria and is also associated with improved DFS and BCSS in univariate but not in multivariate analysis. In conclusion, many controversial findings remain to be elucidated and more work is required to understand the downstream signalling pathways induced by IL-6 and IL-10 to explain the multifunctional roles IL-6/IL-10 play in BC.

## Electronic supplementary material

Below is the link to the electronic supplementary material.

Supplementary material 1 (PDF 397 KB)

## Abbreviations

*BC*: Breast cancer
*BCSS*: Breast cancer-specific survival
*DAB*: Diaminobenzidine
*DBS*: Donor bovine serum
*DFS*: Disease-free survival
*EGF*: Epidermal growth factor
*ER*: Oestrogen receptor
*hMEC-1*: Human microvascular endothelial cells
*hTERT-LEC*: Human telomerase reverse transcriptase immortalised lymphatic endothelial cells
*IHC*: Immunohistochemistry
*IL*: Interleukin
*KM*: Kaplan–Meier
*LVI*: Lymphovascular invasion
*M1s*: Type-1 proinflammatory macrophages
*M2s*: Type-2 anti-inflammatory macrophages
*NPI*: Nottingham Prognostic Index
*PgR*: Progesterone receptor
*REMARK*: Reporting recommendations for tumour marker prognostic studies
*rIL*: Recombinant interleukin
*STAT3*: Signal transducer and activator of transcription 3
*TMA*: Tissue microarray
*TN*: Triple negative
