# Supplementary material for: IL-6 and IL-10 are associated with good prognosis in early stage invasive breast cancer patients
Source: Cancer Immunol Immunother. 2017 Dec 18;67(4):537–49. doi: 10.1007/s00262-017-2106-8 (PMC5860102; doi:10.1007/s00262-017-2106-8)
Supplement: Supplementary file 1 — Supplementary material 1 (PDF 397 KB) [file 262_2017_2106_MOESM1_ESM.pdf]

Supplementary Table 1. Multivariate Cox regression analysis of IL-6/IL-10 expression adjusted to other prognostic factors.

Variables	DFS (IL-6)			BCSS (IL-6)			DFS (IL-10)		
	**HR**	**95.0% CI**	**p-value**	**HR**	**95.0% CI**	**p-value**	**HR**	**95.0% CI**	**p-value**
**IL**	0.788	0.603-1.029	0.08	0.852	0.627-1.157	0.305	0.799	0.543-1.175	0.254
**Age**	0.864	0.588-1.271	0.459	1.206	0.789-1.878	0.397	0.673	0.436-1.037	0.073
**Tumour size**	1.155	1.007-1.325	**0.039**	1.13	0.941-1.148	0.479	1.344	0.956-1.890	0.089
**Tumour stage**	2.862	2.039-4.017	**0.001**	2.785	1.897-3.795	**<0.001**	2.661	1.765-4.012	**<0.001**
**Tumour grade**	1.294	0.984-1.701	0.065	1.635	1.169-2.209	**0.004**	1.097	0.789-1.524	0.581
**NPI**	0.76	0.516-1.120	0.165	1.023	0.713-1.611	0.925	1.022	0.624-1.674	0.932
**ER (-/+)**	1.305	0.896-1.900	0.165	1.782	1.221-2.646	**0.003**	1.329	0.845-2.090	0.219
**PgR (-/+)**	0.869	0.635-1.190	0.382	0.592	0.420-0.817	**0.002**	1.048	0.714-1.538	0.811
**HER-2 (-/+)**	1.205	0.858-1.692	0.281	1.452	1.021-2.073	**0.039**	1.683	1.157-2.448	**0.006**
**LVI (-/+)**	1.491	1.172-1.898	**0.001**	1.493	1.137-1.940	**0.003**	1.762	1.344-2.311	**<0.001**
**LN status**	0.547	0.363-0.822	**0.004**	0.671	0.433-1.040	0.074	0.439	0.270-0.714	**0.001**

Abbreviation: HR, hazard ratio; CI, confidence interval; NPI is Nottingham Prognostic Index; ER, oestrogen receptor; PgR, progesterone receptor; HER-2, epidermal growth factor receptor 2; LVI, lymphovascular invasion; LN, lymph node.

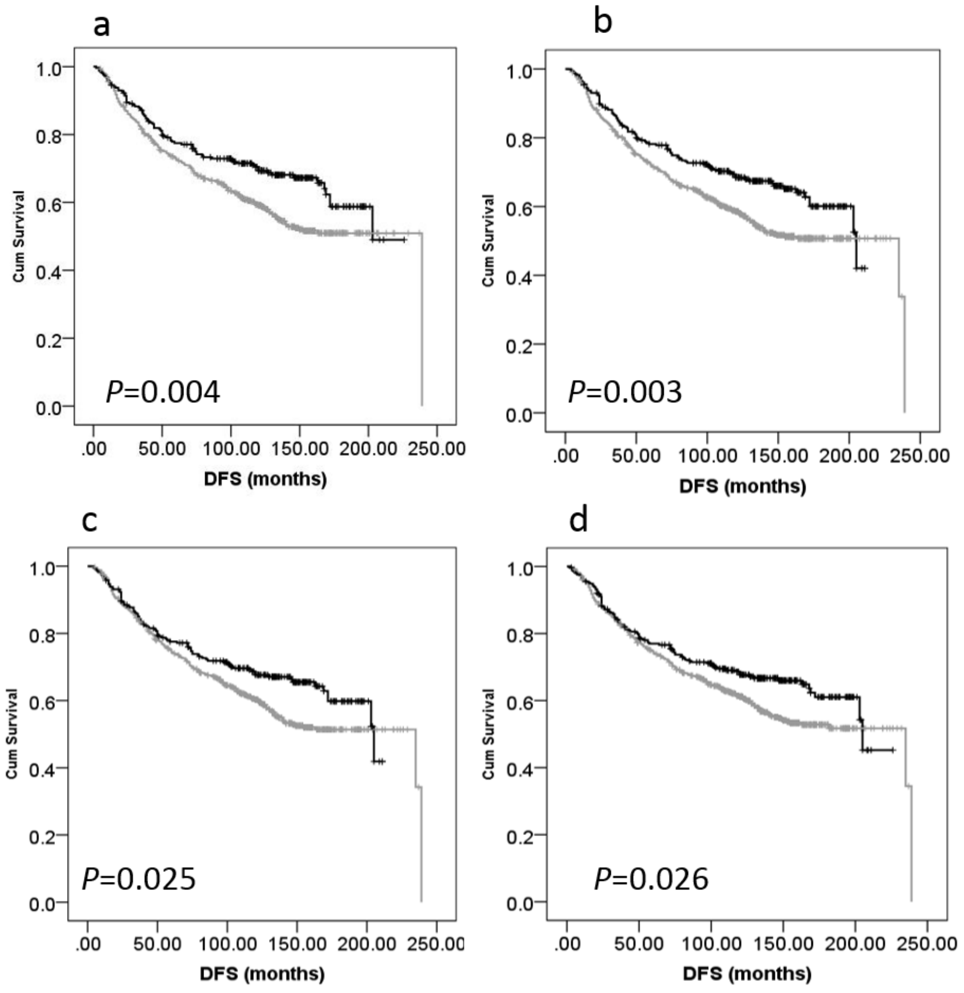

Supplementary Figure 1. Kaplan-Meier analyses of associations of IL-6 expression with disease-free survival in phenotypic subgroups. High IL-6 expression was significantly associated with disease-free survival in: (**a**) non-basal (*P*=0.004), (**b**) non-triple negative (*P* =0.003), (**c**) ER positive (*P*=0.025), and (**d**) HER-2 negative (*P*=0.026) patient subgroups. Significance was determined using the log-rank test. Black represents high expression and grey represents low expression of the cytokine

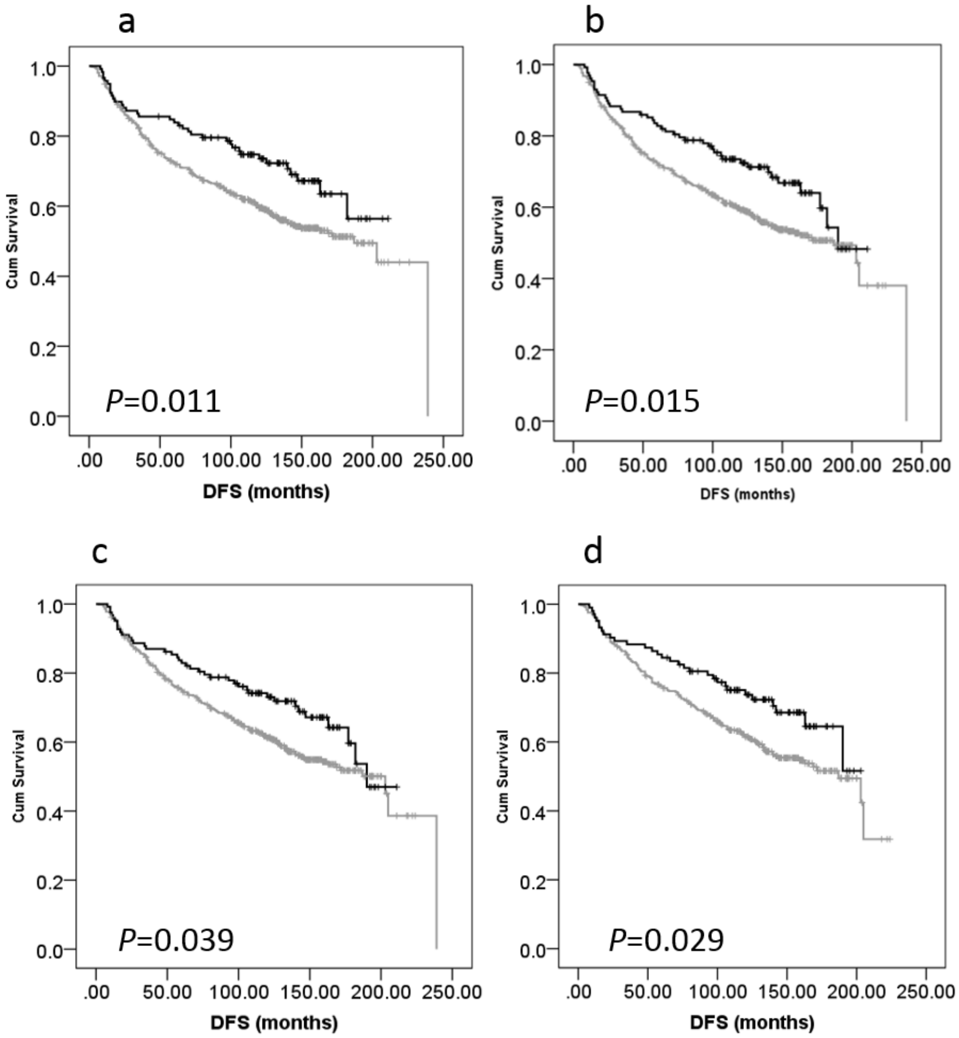

Supplementary Figure 2. Kaplan-Meier analyses of associations of IL-10 expression with disease-free survival in phenotypic subgroups. High IL-10 expression was significantly associated with disease-free survival in (**a**) non-basal (*P*=0.011), (**b**) non-triple negative (*P*=0.015), (**c**) ER positive (*P*=0.039), and (**d**) PgR positive (*P*=0.029) patient subgroups. Significance was determined using the log-rank test. Black represents high expression and grey represents low expression of the cytokine

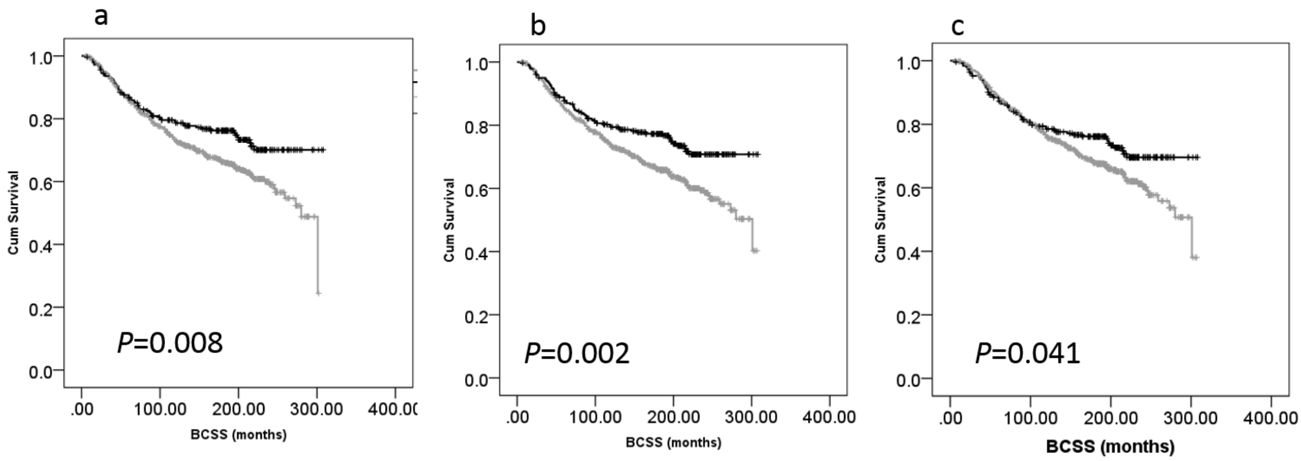

Supplementary Figure 3. BCSS of IL-6 in disease subgroups. Kaplan-Meier BCSS analysis showing the impact of IL-6 in phenotypic subgroups with significance determined using the log-rank test. High IL-6 expression was significantly associated with BCSS in: (**a**) non-basal-like (*P*=0.008), (**b**) non triple negative (*P* =0.002), and (**c**) ER positive (*P* =0.041) patient subgroups. Black represents high expression and grey represents low expression of the cytokine
